# Modulation of orexigenic and anorexigenic peptides gene expression in the rat DVC and hypothalamus by acute immobilization stress

**DOI:** 10.3389/fncel.2014.00198

**Published:** 2014-07-18

**Authors:** Fatiha Chigr, Fatima Rachidi, Catherine Tardivel, Mohamed Najimi, Emmanuel Moyse

**Affiliations:** ^1^Life Sciences, Biological Engineering, Faculty of Sciences and Techniques, Sultan Moulay Slimane UniversityBeni Mellal, Morocco; ^2^Faculté des Sciences et Techniques, Aix-Marseille Université, PPSNMarseille, France; ^3^Biologie Animale et de Génétique, Université François, Rabelais, INRATours, Nouzilly, France

**Keywords:** stress, psychological, POMC, CART, AgRP, NPY, hypothalamo-hypophyseal system, DVC

## Abstract

We studied the long term effects of a single exposure to immobilization stress (IS) (1 h) on the expression of anorexigenic (Pro-opiomelanocortin: POMC and cocaine amphetamine related transcript: CART) and orexigenic (neuropeptide Y:NPY, Agouti related peptide: AgRP) factors in hypothalamus and dorso vagal complex (DVC). We showed, by using RT-PCR that in the hypothalamus, that the mRNAs of POMC and CART were up-regulated at the end of IS and up to 24 h. This up regulation persists until 48–72 h after IS for CART only. In the DVC, their expressions peak significantly at 24 h post stress and decline afterwards; CART mRNA is down regulated after 48 h post stress. NPY and AgRP mRNAs show a gradual increase just after the end of IS. The up regulation is significant only at 24 h after stress for AgRP but remains significantly higher for NPY compared to controls. In DVC, the mRNAs of the two factors show generally a similar post stress pattern. A significant increase jut after the end of IS of rats which persists up to 24 h after is firstly noticed. The levels tend then to reach the basal levels although, they were slightly but significantly higher up to 72 h after stress for mRNA NPY. The comparison between the expression profiles of anorexigenic and the two orexigenic peptides investigated shows the presence of a parallelism between that of POMC and AgRP and that of CART and NPY when each brain region (hypothalamus and DVC) is considered separately. It seems that any surge in the expression of each anorexigenic factor stimulates the expression of those of corresponding and appropriated orexigenic one. These last reactions from orexigenic peptides tend to attenuate the anorexigenic effects of CART and POMC and by consequent to abolish the anorexia state generated by stress.

## Introduction

Stress is well established as a serious health problem in industrialized human societies, since it favors several major pathologies such as cardiovascular failure (Manni et al., 2008; Wirtz et al., 2008), cancer (Quick et al., 2008) and depression (Chaplin et al., 2008). Other emerging deleterious effects of stress include feeding disorders and body weight (BW) dysregulations (Harris et al., 2002; Hamer and Stamatakis, 2008). In animals, many studies reported that different paradigms of stress produce a significant reduction in food intake (FI), unless access to food is given after the stress period. In human, psychological stress paradigms are more effective in producing such manifestations (Vallès et al., 2000; Charrier et al., 2006; Adam and Epel, 2007; Laurent et al., 2013).

FI regulation in adult Mammals is integrated mainly by two brain structures: the hypothalamus and the dorsal vagal complex (DVC), and involves an array of neuroendocrine communications (Schwartz et al., 2000; Berthoud et al., 2006; Morton et al., 2006). Short-term regulation, which consists in reflex arrest of FI under stomachal filling or satiety reflex, is triggered by vagus nerve afferents to the DVC. Long-term regulation consists essentially in satiety reflex threshold modulation by “adiposity signals”, i.e., the peripheral hormones signaling metabolic storage levels like leptin or insulin, which involves the hypothalamus and its reciprocal projection on the DVC (Houpt, 2000; Schwartz et al., 2000; Morton et al., 2006). In the hypothalamus, adiposity signals are integrated into the balance between two mutually antagonistic pathways from the arcuate nucleus: the orexigenic neurons expressing neuropeptide Y (NPY) and the agouti-related peptide (AgRP) as co-neurotransmitters, and the anorexigenic neurons emitting pro-opiomelanocortin (POMC)-derived alpha-melanocyte stimulating hormone (α-MSH) and cocaine and amphetamine regulated Peptide (CART; Schwartz et al., 2000; Cone, 2005; Morton et al., 2006). Both neuron sub-populations are receptive to leptin and display signal-dependent plasticity (Bouret et al., 2004; Pinto et al., 2004).

The receptors and downstream effectors of the orexigenic and anorexigenic neuromediators, like brain-derived neurotrophic factor (BDNF), as well as receptors of leptin and other metabolically relevant systemic messengers, are expressed in specific neuronal populations of the DVC (Cone, 2005). Their involvement in FI regulation is hardly known, except for BDNF (Bariohay et al., 2005; Charrier et al., 2006) and melanocortinergic signaling (Ellacott et al., 2006). Such redundancy supports a distributed, rather than hierarchical model of brain center involvement in feeding regulation (Berthoud et al., 2006; Lebrun et al., 2006). However, actual involvement of DVC peptidergic signaling in FI regulation remains largely unaddressed. Anorexigenic challenges by cholecystokinin or leptin were shown to elicit parallel changes of anorexigenic BDNF expression in DVC and hypothalamus (Fan et al., 2004; Bariohay et al., 2005; Ellacott et al., 2006). Conversely, anorexia-inducing immobilization stress (IS) triggered different BDNF recruitment patterns between DVC and hypothalamus (Charrier et al., 2006). Furthermore, in the framework of hypothalamus-DVC relationships, the precise mechanism by which stress affects energy metabolism as well as FI and BW control is not well understood, notably at the gene expression level. Furthermore, stress could have long lasting effects, depending on the paradigm used. The post stress effects, of acute stress, are less well known, however emerging evidence shows that it can impact later, depending on stress paradigm (Vallès et al., 2000; Charrier et al., 2006). Thus, to further understand the interplay between the central appetite-stimulating and stress signals in the regulation of FI, we examined the post stress dynamics of NPY, AgRP, POMC and CART gene expressions in microdissected hypothalami and DVC from adult rats submitted to immobilization stress.

## Materials and methods

### Animals

**Figure 1:**
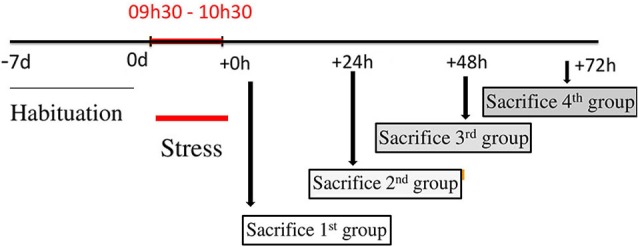
**Design of stress procedures**. This figure represents a schematic presentation with a time-line of treatment schedule. Before stress procedure, a period of habituation is planned to minimize any external influences. Food intake (FI) and body weight (BW) for each rat, were monitored daily during this period. After, 20 rats were immobilized for 1 h and divided into four groups. The first group was sacrificed just after the cessation of stress and corresponds to 0 h group. The second, the third and the fourth groups were sacrificed at 24, 48 and 72 h after the end of immobilization respectively and correspond to 24, 48 and 72 h groups. FI and BW are monitored just before the decapitation for each group.

Twenty-five male Wistar rats (Charles River, Les Oncins, France) weighing 220–250 g were used in this study. The rats were divided into five groups, four stressed groups (*n* = 5 for each group) and a control group (*n* = 5). The four stressed groups consisted of rats sacrificed just after the termination of stress (0 h), 24, 48 and 72 h following the termination of 1 h immobilization. All the rats were housed under a 12:12 h dark:light cycle and constant temperature with water and standard rat chow (pellets A04, Scientific Animal Food and Engineering, Augy, France) *ad libitum*. Animals were handled and cared for in accordance with the *Guide for the Care and Use of Laboratory Animals* (NRC, 1996) and the European Communities Council Directive of 24 November 1986 (86/609/EEC). Experimental protocols were carried out in compliance with institutional Ethical Committee guidelines for animal research. Rats were housed 3–6 per cage, and allowed to acclimatize for at least 5 days after their arrival. All animals were then handled daily for 5 days before the experiment. Some rats were subjected to acute immobilization stress according to a well-established protocol (Kvetnansky and Mikulaj, 1970; Benyassi et al., 1993): each rat was attached on a wooden board in prone position by taping their limbs and shoulders to metal mounts at 9:30 a.m. for 60 min, and returned to its cage. Rats were sacrificed by decapitation at 0, 24, 48 or 72 h after stress; control rats were sacrificed at identical times of day (Figure 1). Tissue blocks of DVC and hypothalamus were microdissected from each rat brain, dropped in sterile tubes and immediately frozen in liquid nitrogen. BW of each animal was measured daily, beginning 1 week before stress and up to sacrifice.

### Control of food intake

FI was measured daily both in controls and stressed animals. Rats were given in excess a known quantity of chow and the amount of food eaten was calculated by subtracting the remaining amount of chow from the original amount provided to the rats. Chow spillage was carefully calculated and accounted for in the measurements. The measurements were made just before stress, 24, 48 and 72 h after stress.

### Control of body weight gain

After 1 week of accommodation, experimental animals were assigned to *ad libitum* feeding, BWs of both control and rats destined to be stressed, were assessed daily, 72, 48 and 24 h before stress application. The BWs of all rats were measured at the same time of the day, and just before the stress session on the day of immobilization stress, 24, 48 and 72 h after stress.

### Fecal pellet

The number of fecal pellets production during immobilization session has been counted and compared to production in controls.

### RNA extractions

Total RNA were extracted from different tissue samples obtained from hypothalamus and DVC. Each frozen tissue block was homogenized on ice in 500 μL TRIzol^*R*^ reagent (Life Technologies, Cergy-Pontoise, France) and RNA was extracted according to the manufacturer’s instructions. Extracted RNA was resuspended in 20 μL DEPC-treated distilled water. RNA concentration and purity were evaluated by spectrometry at 260 and 280 nm on 1 μL aliquots and RNA extracts were kept frozen until use. For protein extraction, each frozen sample was homogenized on ice in 100 μl lysis buffer (137 mM NaCl, 20 mM TrisHCl, 1 mM sodium orthovanadate, 1% Triton X100, 10% glycerol, 1 mM EDTA, 1 mM PMSF, protease inhibitor cocktail, pH 8) and incubated for 30 min on ice. Homogenates were then cleared by centrifugation (10,000 rpm for 30 min at 4°C). Proteins were quantified using the Bradford assay (BioRad, Marnes-la-Coquette, France), and each sample was adjusted to 1 μg/μl with ultra-pure water, mixed to equal volumes of 2X Laëmmli buffer. Extracted proteins were denatured by heat at 70°C for 3 min and then kept frozen until use.

### RT-PCR

Single-stranded cDNAs were synthesized using 2 μg of total RNA of tissue samples by reverse transcription in a total volume of 20 μL using 200U of M-MLV reverse transcriptase (Promega, Madison, WI, USA), 5 μM random hexanucleotide (Promega), 0.2 mM dNTP (Amersham, Saclay, France), and 1X manufacturer’s RT buffer. Specific genes were amplified in a 20 μL reaction mixture containing 2.5 U/μL Taq polymerase (Promega), 0.1 μM dNTP (Amersham), 1.5–2.5 mM MgCl_2_ (see Table 1), 1 μM specific primer pair (Invitrogen, Cergy-Pontoise, France, Table 1) 1X manufacturer’s Taq buffer, and 1 ul cDNA through a PCR program including one DNA denaturation step (4 min at 95°C), 30–35 cycles consisting of DNA denaturation (45 s at 95°C)—primer hybridization (30 s at optimal temperature)—elongation (1 min 30 s at 72°C), and a final elongation step (10 min at 72°C). Primers and PCR program used in the amplification of genes are presented in Table 1. PCR products were separated by electrophoresis on 1% agarose gel stained with ethidium bromide and then visualized under UV light connected to a computer-assisted analyzer (GelDoc, Biorad, Marnes-la-Coquette, France). Optical densities (OD) of PCR products were measured and normalized to OD values from β-actin PCR of the corresponding cDNAs.

**Table 1:** **PCR parameters for amplification of NPY, AgRP, POMC, CART mRNAs under basal conditions**.

**Transcript**	**Sequence (3′-5′) forward (f) ad reverse (r)**	**Product size (bp)**	**[Mg^2+^] (mM)**	**Annealing temperature**	**Number of cycles**	**Genbank accession number**
NPY	f: gcc atg atg cta ggt aac aaa r: tct ctt gcc ata tct ctg tct	214	1.5	55°C	30	*M20373*
AgRP	f: gct gca gaa ggc aga agc r: tga aga agc ggv agt agc ac	152	1.5	58°C	35	AF206017
POMC	f: cgg ccc cag aaa cag cag cag t r: ggg ccc gtc ggc ctt ctc g	304	1.5	66°C	30	NM_139226
CART	f: gat cgg gaa gct gtg tga ct r: att ttg aag cag cag gga aa	208	1.5	56°C	31	NM_017110

### Statistical analysis

The results are expressed as mean ± standard error of the means (S.E.M). The results were analyzed by one-way ANOVA followed by post-hoc analysis using Tukey tests. *p* < 0.05 was considered statistically significant.

## Results

### Effects of 1 h immobilization on food intake

**Figure 2:**
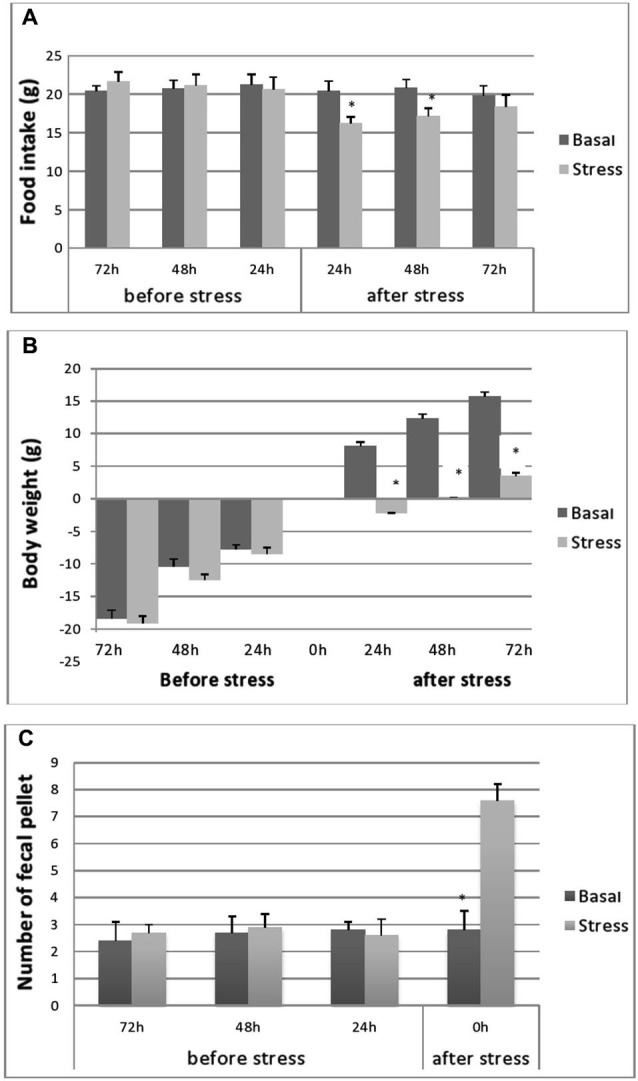
**Cumulative food consumption (g) before and after stress (A), body-weight evolution (B) of stressed and control rats during the pre- and post-stress period and fecal pellet production.** **(C)** Fecal pellet production before and during stress. Food consumption and BW evolutions were monitored 72, 48 and 24 h before stress and 24, 48 and 72 h after stress. FI in controls was processed at same points as stressed rats. Data are represented as the mean ± S.E.M. Differences between controls and stressed rats are significant at each experimental point after immobilization session (*p* < 0.001) (*n* = 5 per group) for FI and BW evolutions and *p* < 0.001 for fecal pellets number.

Cumulated daily FI was reduced significantly following 24 h after the cessation of stress (16.2 ± 0.8 *vs*. 20.4 ± 1.3, *p* < 0.001; Figure 2A). This relative hypophagia in stressed rats was no longer observed after 48 h of stress (Figure 2A).

### Effects of 1 h immobilization on rat’s body weight

As depicted in Figure 2B, no differences were seen in BW gain levels between unstressed and stressed rats, over the course of the period preceding the experiment. Both groups of rats gained weight at a steady rate. However, as expected, during the 24 h following the stress application, the stressed animals showed a significant decrease in their BW gain (−2.2 ± 0.7 g; *n* = 5; *p* < 0.001) compared with the important BW gain recorded for control rats (8.1 ± 0.6 g; *n* = 5). However, at 48 h post stress period, a slight increase in BW was observed in stressed animals but below control values (Figure 1B).

### Effects of stress on fecal pellets

Fecal pellet output (i.e., the number of fecal pellets produced) during each stress session was significantly higher in immobilized rats (7.25 ± 1.01) compared to controls (2.59 ± 0.66) (*p* < 0.01) (Figure 2C).

### Different regulation of orexigenic and anorexigenic peptides

**Figure 3:**
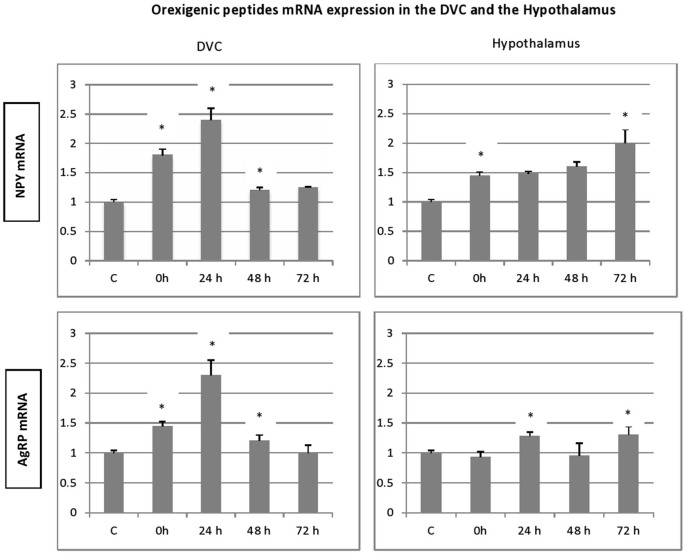
**Time-course and dynamic changes of the effects of 1 h stress immobilization session on profiles in hypothalamic NPY mRNA, AgRp mRNA, POMC mRNA and CART mRNA (data from 6–11 rats/time point)**. * significantly different from control rats. *p* < 0.05.

**Figure 4:**
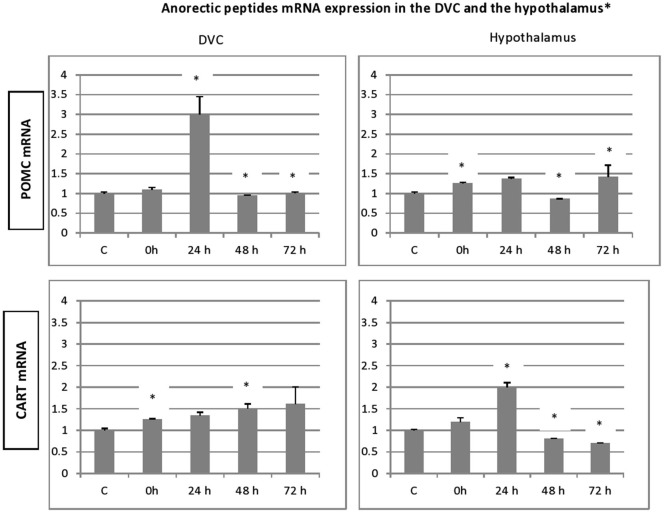
**Time-course and dynamic changes of the effects of 1 h stress immobilization session on profiles in DVC NPY mRNA, AgRp mRNA, POMC mRNA and CART mRNA (data from 6–11 rats/time point).** * significantly different from control rats. *p* < 0.05.

To evaluate eventual variations in the expression of genes encoding the different peptides after stress exposure, their levels were determined relative to that of β-actin mRNA. In all groups, significant amounts of each peptide (NPY, AgRP, POMC and CART) mRNA were detected in the adult rat hypothalamus (Figure 3). Furthermore, we show the presence of important levels of peptides mRNA in the microdissected DVC of adult rat (Figure 4).

In general, the effect of stress on the expression level of four peptides, showed similar patterns especially, in the hypothalamus. A significant increase in gene expression was observed for hypothalamic NPY and CART gene. This increase was observed immediately after the termination of the stress session and up to 72 h post stress. However, the NPY transcript levels in stressed animals were not significantly different from the controls (Figure 3). Hypothalamic POMC mRNA profile mimics partially the two precedent patterns, a significant increase in POMC transcript was observed up to 24 h post stress (Figure 3; *p* < 0.05). Finally, the AgRP expression increased significantly at 24 h and to a lesser extent at 72 h post stress (Figure 3; *p* < 0.05).

With regard to DVC, a general similar pattern post stress evolution of the mRNA peptides level was observed for the peptides analyzed. A substantial and significant increase was observed particularly at 24 h post stress, and a decline in the up regulation was noticed at 48 and 72 h post stress. A striking similarity between the patterns of expression of the genes encoding the two orexigenic peptides was observed (Figure 3). The mRNA of both NPY and AgRP increase significantly just after the end of 1 h immobilization session and the increase became more important at 24 h after stress (Figure 3; *p* < 0.01). In contrast to NPY and AgRP, the expression of POMC failed to increase after the end of stress but showed a strong and significant elevation at 24 h, the highest one of the four peptides analyzed (Figure 4; *p* < 0.01). CART expression respects the general post stress mRNA peptides evolution in that it increases at 0 and 24 (*p* < 0.01 at 24 h). However, at 48 and 72 h post stress period, the profile differs from that observed for the other peptides. Indeed, there was a clear and significant down regulation of CART expression compared to NPY, AgRP and POMC (Figure 4; *p* < 0.01 at 48 h and *p* < 0.001 at 72 h).

## Discussion

The aim of the present study was to evaluate if acute immobilization stress in adult rat has long lasting effects on FI regulation. The results demonstrated that the application of acute immobilization stress (1 h) increased significantly the expression level of the four peptides studied in the hypothalamus and DVC regions. Both orexigenic (NPY and AgRP) and anorexigenic (CART and POMC) genes were up regulated principally at 24 h post stress. Concomitantly, acutely immobilized rats showed a substantial decrease in BW and FI, especially at 24 h post stress. These two parameters were monitored in order to determine whether these FI indicators were related to the expression of the genes encoding the orexigenic and anorexigenic peptides. Taken together, the differences in signaling and behavior suggest that systems involved in the regulation of the stress response and of energy balance are highly integrated. We assessed the post stress period as this phase shows high sensitivity to environmental changes, a long-lasting sensitisation of the hypothalamo-pituitary-adrenal (HPA) axis and corresponds to the recovery period (Dal-Zotto et al., 2004). The present observations showing significant variations in the tissue contents of orexigenic and anorexigenic peptides were related specifically to acute immobilization stress. Indeed, the protocol schedules we used have minimized the effects of handling and eliminated any other artifacts by standardizing the experiments before brain dissection.

Our results showed that 1 h immobilization stress affects differentially the amplitude and the direction of gene expression changes depending on the post period point and the brain region investigated, although in all cases, an up regulation of the gene expression of each peptide was observed 24 h after the cessation of stress session. This may indicate that at this experimental point corresponding to the “day after”, profound changes occur in the regulatory control of FI, in support of this assertion the marked decrease in BW gain and FI amount recorded. Nevertheless, hypothalamus and DVC display different responses to stress. The hypothalamus is more sensitive to stress effect as just after the termination of immobilization and except for AgRP, the gene expressions of NPY, POMC and CART are up regulated. In DVC, only the expressions of orexigenic peptides investigated are up regulated. This stress sensitivity in hypothalamus could be explained at least in part by the mobilization of the HPA axis notably via the actions of glucocorticoids (GCs). This system also regulates feeding responses as the neural circuits regulating FI converge on the PVN containing corticotrophin releasing hormone (CRH) neurons (the primary target for initiating a stress response). CRH is not only the major regulator of pituitary-adrenal axis, but it is also involved in energy homeostasis, processing catabolic effects, restraining FI and activating sympathetic nervous system (Fulton et al., 2002; Guillemin, 2005). Interestingly, the interaction between GCs and feeding related neuropeptides such as NPY, CART, AgRP and POMC has been well documented (Savontaus et al., 2002; Germano et al., 2007). Thus, a surge of GCs results in an increase in the expression of these neuropeptides at least in hypothalamus. Furthermore, the presence of GC receptors within NPY/AgRP and POMC/CART neurons suggest a direct role of corticosterone on these neurons. It is known that GCs levels rise at 15 min after the beginning of the immobilization stress (Rage et al., 2002; Marmigère et al., 2003) and this peak could be sufficient to stimulate the expression of peptides during or at the end of stress session giving an explanation to the rise of gene expression observed. The delayed effect on AgRP gene expression could be a result of differential sensitivity of AgRP neurons although, co-localized exclusively with NPY in the arcuate nucleus (Broberger et al., 1998). AgRP is more important during conditions of high energy requirements under which it has been shown to be more highly expressed (Sorensen et al., 2002) as in the period of 24 h post stress, where an anorexia-like state has been occurred. Of interest, stimulation of AgRP neurons profoundly increased FI and decreased energy expenditure (Aponte et al., 2011), leading to a recovery of control conditions. Furthermore, stimulation of AgRP in the absence of food increased locomotor activity indicating that the neuropeptide could be involved in the regulation of motivations associated with food-seeking behaviors (Sohn and Williams, 2012). Another alternative explanation is the possible involvement of the melanocortin pathway as it has been shown that AgRP suppression of POMC-derived melanocortin signaling has been widely considered as a pathway through which these neurons could rapidly increase feeding behavior (Cowley, 2003). Thus, the increase of DVC AgRP gene expression just after the termination of stress up to 24 h before POMC gene expression suggests that AgRP system drive the expression of the POMC to attenuate the ongoing established anorexia state. Another discrepancy between hypothalamus and DVC is illustrated by the post-stress pattern of gene expressions in the two structures. Whereas they show a marked peak in the DVC at 24 h post stress followed by a gradual decrease, they display a gradual increase up to 72 h post stress in the hypothalamus, particularly for NPY and CART expressions. This could reflect a stress neuroendocrine regulation as we stressed above about GCs action on these two neuropeptides which in turn have been reported to stimulate CRF and increase plasma ACTH and corticosterone following stress (Suda et al., 1993; Stanley et al., 2001). This last situation underlies behavioral adaptation, storage of energy and information processing to prepare for future events (De Kloet, 2004). The increase in NPY expression may be also a response to the anxiogenic-like effects on stress response (Kim et al., 2003) as the peptide is negatively correlated with anxiety symptoms (Heilig et al., 1989). In this sense, NPY will act as a stress buffer to counteract anxiogenic effects of stress (Heilig et al., 1994). Of interest, single intranasal NPY infusion attenuates development Post-Traumatic Stress Disorder: PTSD-like symptoms to traumatic stress in rat (Serova et al., 2013). The fact that the magnitude of up regulation is more marked in hypothalamus than in the DVC, could be explained by the occurrence of high peptide containing neurons levels in the hypothalamus compared to the brainstem structure (Heilig and Widerlöv, 1995), although these project to the CRH hypothalamic paraventricular neurons (Liposits et al., 1988). Taken together, the resemblance of NPY expression responses observed in these two structures could indicate that the severe physiologic stress used in these investigations appears to trigger brainstem/circumventricular organ systems that project directly to the paraventricular nucleus.

With regard to CART, an equivalent pattern to that observed for NPY gene expression is present in hypothalamus. This continuous vigorous over-expression could reflect a strong response of the anorexigenic peptide to stress (notably via their stimulation by GCs), establishing an anorexia-like state by inhibiting FI. An elevation in hypothalamic CART gene expression has also been reported to coincide with the onset of short day anorexia in hibernating Siberian hamster (Adam et al., 2000). In line of this, central administration of CART has been reported previously to inhibit FI (Vrang et al., 1999). For this, it would seem logical that the actions of CART following stress are anorectic and trigger in turn equivalent reaction of NPY to counteract these effects. On the other hand, it is noteworthy that important increase in CART expression (48–72 h post stress) is associated with a recovery of normal BW and FI suggesting the involvement of the peptide in energy homeostasis (Smith et al., 2008) and possible orexigenic role (Abbott et al., 2001; Kong et al., 2003). This passes via an interaction with CB1 receptors (Cota et al., 2003; Yu et al., 2008). With regard to DVC signaling responses following stress, peptide expression pattern differs significantly from that observed in the hypothalamus. Stress leads to a down regulation observed at 48 h post stress time following a rapid up regulation period, which may suggest that acute CART induction might trigger immobilization-induced anorexia principally at the hypothalamus, as a FI regulation center and at a lesser degree at the satiety-reflex-integrating DVC. This phase of down regulation could correspond to the inhibition of a tonic satiety-enhancing mechanism as suggested for another anorexigenic peptide the BDNF (Lebrun et al., 2006). Therefore, immobilization-induced modulations of CART expression in hypothalamus and DVC would not be a downstream effect of peripheral stress response, but would more likely result from an intracerebral pathway upstream of the neuroendocrine axis, which seems in accordance with the psychological quality of immobilization.

It is certain that NPY and CART interact with other factors to modulate stress responses. In this sense, it is interesting to note that expressions of AgRP (an orexigenic peptide) and POMC (an anorexigenic peptide) were also up regulated following immobilization in the hypothalamus although they do not exceed 24 h post stress. Thus, it is not excluded that AgRP could potentates NPY action as POMC could do to CART action. These concomitant actions in anorexigenic and orexigenic peptides could contribute to the fine tuning of homeostasis regulation. Thus, any deregulation of these actions, by an excess simultaneous secretion of the components or a non adequate integration of NPY and AgRP signals (Kas et al., 2005) for example, could lead to a blurred signal which could be fatal during the recovery period and may lead to an irreversible pathological state as the installation of anorexia nervosa (Inui, 2001).

In summary, differences in neuropeptides expression in response to stress could reflect gene function regulation as adaptative behavioral responses to environmental events. Any disturbance at this level could contribute to the development of eating disorders. It remains to be investigated whether similar changes will be observed at the protein level and neuropeptide release.

## Conflict of interest statement

The authors declare that the research was conducted in the absence of any commercial or financial relationships that could be construed as a potential conflict of interest.
